# Targeted Therapies in Axial Psoriatic Arthritis

**DOI:** 10.3389/fgene.2021.689984

**Published:** 2021-06-28

**Authors:** Alberto Floris, Mattia Congia, Elisabetta Chessa, Maria Maddalena Angioni, Matteo Piga, Alberto Cauli

**Affiliations:** Unità Operativa Complessa di Reumatologia, Dipartimento di Scienze Mediche e Sanità Pubblica, Azienda Ospedaliero-Universitaria e Università di Cagliari, Monserrato, Italy

**Keywords:** psoriatic arthritis, targeted therapy, spondylarthritis, axial psoriatic arthritis, treatment

## Abstract

Specific and high-quality evidence on the efficacy of the current targeted therapies for axial disease in psoriatic arthritis (axPsA) is still scarce. Indeed, almost all the cohorts investigated in clinical trials on PsA consisted of patients with peripheral arthritis, where a small number of them also had axial involvement. Only one randomized controlled trial was so far specifically designed to assess the efficacy of a biological disease-modifying antirheumatic drug (DMARD) in axPsA. For other biological and synthetic targeted DMARDs, the most specific evidence for treatment in axPsA is extrapolated from *post-hoc* analyses based on PsA patients with concomitant peripheral and axial manifestations. Furthermore, the current trials and *post-hoc* analysis on axPsA are affected by major limitations, including the lack of a widely accepted definition of axPsA and the lack of specific and validated outcome measures. Finally, poor data are available on the genetics of axPsA, although alleles differentially expressed in different patterns of axPsA might offer advantages in the prospective of personalized medicine in axPsA patients. Overall, this review suggests that there is an urgent need for more reliable evidence derived from studies specifically designed for axPsA and based on a validated definition of axPsA and on specific outcome measures.

## Introduction

Psoriatic arthritis (PsA) is a heterogenous systemic inflammatory disease with different subtypes of joint manifestations, including peripheral arthritis, dactylitis, enthesitis, and axial disease (Moll and Wright, 1973). The prevalence of axial disease in PsA (axPsA) widely varies depending on its definition and disease duration (Gladman, 2007). Indeed, isolated spondylitis was described by Moll and Wright in only 5% of patients with PsA (Moll and Wright, 1973), but when axial involvement is defined by inflammatory back pain and/or radiography along with peripheral involvement, its prevalence ranges from 5 to 28% in early PsA to 25–70% in longstanding PsA (Gladman, 2007; Chandran et al., 2009).

Despite the relatively high occurrence of axPsA, several unresolved issues persist with regard to its definition, classification, and management (Gladman et al., 2021). An evidence-based and widely accepted definition of axPsA does not exist yet (Feld et al., 2018). Furthermore, although significant epidemiological clinical and prognostic differences between axPsA and ankylosing spondylitis (AS) have been highlighted in several studies (Jadon et al., 2017; Feld et al., 2018), it is still a matter of debate whether axPsA and AS are different phenotypes on the spectrum of the same disease, or they are different diseases with overlapping features (Feld et al., 2018). Genetic studies were called into question to understand the basis of such differences and overlapping aspects (Rahmati et al., 2020). In particular, the prevalence of HLA-B27, the key genetic marker of AS, was demonstrated to be lower in patients with PsA (20 vs. 95%), although among PsA patients, those with axPsA were significantly more frequently HLA-B27-positive compared with those without axial involvement (*P* < 0.001). Moreover, when different patterns of axial involvement were separately investigated, HLA-B27 was recorded in only 22% of PsA patients with unilateral sacroiliitis, which is more typical in axPsA, compared with 85% of patients with bilateral sacroiliitis (Vecellio et al., 2021). Conversely, in a study on 282 PsA patients, HLA-B^*^0801 was significantly more frequent in patients with asymmetrical sacroiliitis (Winchester et al., 2016).

In this context of uncertainty in the definition and classification of axPsA, the current recommendations for the management of PsA patients with axial involvement are mainly extrapolated from studies on AS or non-radiographic axial spondylarthritis SpA (nr-axSpA), and poor specific evidence exists regarding the use of the available biologic (b-) and targeted synthetic (ts-) disease-modifying antirheumatic drugs (DMARDs) in ax-PsA (Gossec et al., 2012; Coates et al., 2016; Singh et al., 2019).

The objective of this work was to summarize current evidence regarding the efficacy of the currently available b- and ts-DMARDs, including tumor necrosis factor-alpha (TNFα) inhibitors, interleukin (IL)-17 inhibitors, IL-23 and IL-12/23 inhibitors, and Janus kinase (JAK) inhibitors, in treatment of axPsA.

### Tumor Necrosis Factor-Alpha Inhibitors

*Infliximab* is a chimeric human–murine monoclonal antibody (MoAb) that binds with high affinity to both soluble and transmembrane forms of TNF (Antoni, C. E. et al., 2005; Antoni, C. et al., 2005). It is approved for the treatment of PsA and AS. In the pivotal randomized controlled trials (RCTs) submitted for its approbation in PsA (IMPACT 1-2), <5% of patients had axial involvement along with peripheral arthritis, and no specific sub-analyses on axial outcomes were performed (Antoni, C. E. et al., 2005; Antoni, C. et al., 2005). No RCTs were specifically implemented in axPsA patients treated with infliximab.

*Etanercept* is a soluble human TNF-α receptor p75 Fc fusion protein that inhibits the biological activity of TNF by competitively binding to its cell surface receptors (Mease et al., 2004). It is licensed for treatment of PsA, AS, and non-radiographic axial spondylarthritis (nr-axSpA). In the RCT submitted for approbation in treatment of PsA, only 3% of patients had axial involvement, and respective outcomes were not analyzed. No RCTs have specifically tested the efficacy of etanercept in axPsA (Mease et al., 2004).

*Adalimumab* is a human immunoglobulin G (IgG)1 MoAb targeting TNF (Mease et al., 2005; Genovese et al., 2007). It is licensed for the treatment of PsA, AS, and nr-axSpA. In the two RCTs (ADAPT, M02-570 Study) submitted for approbation in treatment of PsA, <2% of patients had axial involvement, and axial outcomes were not assessed (Mease et al., 2005; Genovese et al., 2007). No RCTs have been implemented in adalimumab-treated axPsA patients.

*Golimuma*b is a human monoclonal IgG1 antibody that forms high-affinity, stable complexes with both the soluble and transmembrane TNFs (Kavanaugh et al., 2009). It is licensed for the treatment of PsA, AS, and nr-axSpA. In the RCT (GO-REVAL) assessing the efficacy of golimumab in PsA, 10% of patients were reported to have axial involvement; however, no specific definition on the axial disease was provided, and analysis on the outcomes of axial involvement was not assessed (Kavanaugh et al., 2009). No RCTs were specifically implemented in axPsA patients treated with golimumab.

*Certolizumab pegol* is a humanized antibody Fab' fragment against TNF conjugated to polyethylene glycol (Cauli et al., 2015). It is licensed for the treatment of PsA, AS, and nr-axSpA. In the RCT (PsA001) assessing its efficacy in the treatment of PsA, no data were reported on prevalence and outcomes of axial involvement (Mease et al., 2014). No RTCs have specifically assessed the efficacy of certolizumab in axPsA.

Several RCTs demonstrated the efficacy of different TNF inhibitors in reducing clinical disease activity in AS and nr-ax-SpA, as assessed by composite indices, including the Assessment in AS (ASAS) criteria and the Bath AS Disease Activity Index (BASDAI) (van der Heijde et al., 2005; Haibel et al., 2008). Albeit less univocally, efficacy was also recorded in reducing the radiographic progression in these conditions (van der Heijde et al., 2008). No sub-analyses were performed in patients with concomitant psoriasis. A meta-analysis confirmed a significant association between HLA-B27 and a higher rate of response to TNF-α inhibitors in AS, as assessed by the ASAS-40 [odds ratio 1.83, 95% confidence interval (CI) 1.39–2.42] and the BASDAI-50 (odds ratio 1.81, 95% CI 1.35–2.42). Furthermore, with regard to pharmacogenomic, no association was shown between −308 TNF gene polymorphism and BASDAI response (Maneiro et al., 2015).

### Interleukin-17 Inhibitors

*Secukinumab* is a fully human IgG1κ MoAb that selectively binds to IL-17A with high affinity. It is approved for the treatment of both PsA and axSpA.

Of three RCTs (FUTURE 1, 2, and 5) submitted for approval of secukinumab in treatment of PsA, all were conducted in patients with active peripheral arthritis, according to the inclusions criteria, and none provided data on axial involvement (McInnes et al., 2015; Mease et al., 2015; van der Heijde et al., 2020).

A recent phase 3 double-blind, randomized trial evaluated the efficacy and safety of secukinumab in managing axial manifestations in PsA patients, who have failed to respond to non-steroidal anti-inflammatory drugs and were bio-naïve (Baraliakos et al., 2020). Patients treated with secukinumab 300 and 150 mg, when compared with placebo-treated patients, had a significantly higher rate of ASAS-20 (63 and 66 vs. 31%) and ASAS-40 (44 and 40 vs. 12%) achievement at week 12. Furthermore, the least-squares means of treatment difference vs. placebo in change from baseline to week 12 in total Berlin magnetic resonance imaging (MRI) score for the entire spine were −0.4 (0.1) and −0.4 (0.1) for secukinumab 300 and 150 mg, respectively (*p* < 0.05) (Table 1) (Baraliakos et al., 2020).

**Table 1:** Summary of studies specifically designed to assess the effectiveness of different biologic DMARDs in patients affected by psoriatic arthritis with axial involvement.

**Treatment**	**References**	**Definition of axial involvement**	**Type of study**	**N**	**Main findings**
Secukinumab (SEC)	Baraliakos et al., 2020	- Clinician-diagnosed axial involvement - Spinal pain VAS > 40/100 - BASDAI >4 (despite ≥2 NSAIDs)	Phase III double blind RCT (MAXIMASE)	498	•SEC 300 and 150 mg significantly improved ASAS20 response vs. PBO at week 12 (63% and 66 vs. 31%). •SEC 300 and 150 mg significantly improved ASAS40 response vs. PBO at week 12 (44% and 40 vs. 12%). •Least square means (LSM) of treatment difference of SEC 300 and 150 mg vs. PBO from baseline in total Berlin MRI score for the entire spine at week 12 was −0.4 (< 0.001) and −0.4 (*p* < 0.05).
Ixekizumab (IXE)	Deodhar et al., 2019a	- Self-reporting axial pain starting before the age of 45 years at baseline	*Post-hoc* integrated analysis of two phase III RCTs (SPIRIT-P1/P2)	105	•Pain and stiffness significantly improved at Weeks 16 and 24 in patients with PsA treated with IXEQ4W or IXEQ2W vs. PBO (*p* < 0.05). •Fatigue significantly improved at Week 16 in patients treated with IXEQ4W or IXEQ2W vs. PBO and at Week 24 with IXEQ2W vs. PBO (*p* < 0.05). •Total BASDAI scores significantly improved at Weeks 16 and 24 in patients treated with IXEQ4W or IXEQ2W vs. PBO (*p* < 0.01). •Physical function significantly improved at Weeks 16 and 24 in patients treated with IXEQ4W or IXEQ2W vs. PBO when assessed by HAQ-DI or SF-36 PCS (*p* < 0.05).
Ustekinumab (UST)	Kavanaugh et al., 2016	- Clinician-diagnosed spondylitis	*Post-hoc* integrated analysis of two phase III RCTs (PSUMMIT-1 and 2)	256	•At week 24, significantly more patients achieved BASDAI20/50/70 responses (54.8/29.3/15.3% vs. 32.9/11.4/0%; *p* ≤ 0.002). •Higher improvement in BASDAI question 2 concerning axial pain in UST-treated vs. PBO 1.85 vs. 0.24 (*p* < 0.001) and mean per cent ASDAS-CRP improvements (27.8 vs. 3.9%; *p* < 0.001) for UST vs. PBO.
Guselkumab (GUS)	Helliwell et al., 2020	- Imaging-confirmed sacroiliitis	*Post-hoc* integrated analysis of two phase III RCTs (SPIRIT-P1/P2)	312	•In GUS 100 mg q4w, q8w, and PBO groups, the LS mean change at 24w in BASDAI was −2.7, −2.7, −1.3 (*p* < 0.001); in modified BASDAI was −2.6, −2.7, −1.4 (*p* < 0.001), in spinal pain was −2.5, −2.7, −1.4 (*p* < 0.001); in ASDAS was −1.4, −1.4, −0.7 (*p* < 0.001). •The rate of BASDAI 50 achievement was 38, 40, 19% (*p* < 0.01).
Upadacitinib (UPA)	Deodhar et al., 2020a,c	- Clinician-diagnosed spondylitis	*Post-hoc* integrated analysis of two phase III RCTs (SELCET-PsA1-2)	541–626	•Mean delta-BASDAI at week 12: 1–75 and −2.22 in UPA 15 and 30 mg vs. PBO −0.56 (*p* < 0.001). •Mean delta-BASDAI at week 24: −2.61 and −2.71 in UPA 15 and 30 mg vs. PBO −1.00 (*p* < 0.001).

*PBO, placebo; BASDAI, Bath Ankylosing Spondylitis Disease Activity Index; ASAS, Assessment of Spondylarthritis International Society; ASDAS, Ankylosing Spondylitis Disease Activity Score*.

Three RCTs (MEASURE 1, 2, and 3) assessed the effectiveness and safety of secukinumab in AS, but the proportion of patients with PsO was minimal (<10%), and sub-analysis on this subgroup of patients was not performed (Baeten et al., 2015; Pavelka et al., 2020). In a *post-hoc* analysis aimed to assess the impact of HLA-B27 status on clinical outcomes in AS patients treated with secukinumab, it was recorded that secukinumab was effective regardless of HLA-B27 status, although HLA-B27-positive patients may derive increased therapeutic benefit (Deodhar et al., 2020a). In the only RCT (PREVENT) demonstrating the effectiveness of secukinumab in nr-ax-SpA, no data on psoriasis were reported (Deodhar et al., 2021).

*Ixekizumab* is a recombinant IgG4κ MoAb that binds with high affinity to and neutralizes IL-17A. It is approved for the treatment of both PsA and axSpA. Of two RCTs (SPIRIT-P1 and -P2) assessing effectiveness and safety of ixekizumab in the treatment of PsA, all had the presence of peripheral arthritis as an inclusion criterion, and none provided data on the proportion of patients with axial involvement nor assessed axial outcomes (Mease et al., 2017; Nash et al., 2017). A *post-hoc* integrated analysis of these two trials was conducted in a subset of 105 PsA patients with self-reporting axial pain starting before the age of 45 years (axial imaging not included). When compared with placebo, ixekizumab 80 mg every 4 weeks was associated with significantly higher improvement of axial pain (BASDAI question #2 −3.25 vs. −1.26, *p* < 0.001), stiffness (BASDAI question #4/5 −2.5 vs. 0.32, *p* < 0.01), fatigue (−1.84 vs. −0.53, *p* < 0.05), and total BASDAI score (−2.8 vs. 0.78, *p* < 0.01) at 16 weeks. Similar results were recorded at 24 weeks and for ixekizumab every 8 weeks (Table 1) (Deodhar et al., 2019a). Of the three RCTs evaluating the efficacy of ixekizumab in the treatment of AS (COAST-V and -W) nr-axSpA (COAST-X), none reported data on occurrence on concomitant psoriasis (Deodhar et al., 2020b; Dougados et al., 2020). In the *post-hoc* analysis of COAST-X based on HLA-B27 status and disease duration, patients treated with ixekizumab saw improvement in signs and symptoms of nr-axSpA as assessed by ASAS-40 and BASDAI50 responses regardless of HLA-B27 status (positive or negative) or disease duration (<5 or ≥5 years) (Navarro-Compán et al., 2020).

*Brodalumab* is a fully human MoAb that binds to the IL-17 receptor subunit A with high affinity. It has recently been approved for the treatment of PsA. Results from two recently published phase II RCTs (AMVISION 1 and 2) showed a significant improvement in signs and symptoms of PsA in patients treated with brodalumab vs. placebo. However, axial involvement was not assessed (Mease et al., 2021). No RCTs are available in axPsA and axSpA.

### Interleukin-12/23–IL23 Inhibitors

*Ustekinumab* is a fully human MoAb with a high affinity for the p40-subunit shared by IL-12 and IL-23. It is approved for the treatment of moderate to severe psoriasis and PsA but not for axSpA. Two phase 3 RCTs (PSUMMIT 1 and 2) demonstrated the efficacy of ustekinumab in the treatment of multiple domains of PsA, including peripheral arthritis, enthesitis and dactylitis, and radiographic progression of joint damage (Kavanaugh et al., 2016). Approximately 30% of patients from both the trials had physician-reported spondylitis, and ustekinumab demonstrated significant improvements in axial symptoms at week 24, as assessed by the BASDAI, regardless of prior TNF inhibitor use. A *post-hoc* integrated analysis of these RCTs was conducted in 223 PsA patients with peripheral arthritis and physician-reported spondylitis. At week 24, significantly more patients achieved BASDAI 20, 50, and 70 responses (54.8, 29.3, and 15.3 vs. 32.9%, 11.4 and 0%; *p* ≤ 0.002), improvement in BASDAI question #2 concerning axial pain (−1.85 vs. −0.24; *p* < 0.001), and mean percent ASDAS-CRP improvements (27.8 vs. 3.9%; *p* < 0.001) for ustekinumab vs. placebo recipients, respectively (Table 1) (Kavanaugh et al., 2016). On the other hand, three RCTs assessing efficacy and safety of ustekinumab in the treatment of AS and nr-axSpA were discontinued because the primary and major secondary endpoints (ASAS-20, 40, and ASDAS improvement) were not met (Deodhar et al., 2019b).

*Guselkumab* is a human IgG1λ MoAb that binds to the IL-23p19 subunit and inhibits the downstream signaling of IL-23. It is approved for the treatment of PsA but not axSpA. Two RCTs (DISCOVER 1 and 2) demonstrated the efficacy of guselkumab in the treatment of PsA, as assessed by ACR 20, 50, and 70 (Deodhar et al., 2018). A *post-hoc* integrated analysis of these studies was carried out in 312 PsA patients with peripheral arthritis and imaging-confirmed axial involvement consistent with sacroiliitis (both bio-naïve and not). In patients treated with guselkumab 100 mg every 4 weeks vs. placebo, a greater LS mean changes from baseline to week 24 in BASDAI (−2.67 vs. −1.35, *p* < 0.001), spinal pain (BASDAI question #2 −2.73 vs. −1.30, *p* < 0.001), modified BASDAI (−2.16 vs. −1.13, *p* < 0.001), and ASDAS-CRP (1.43 vs. −0.71, *p* < 0.001) were recorded (Helliwell et al., 2020). Moreover, a greater proportion of guselkumab-treated patients achieved BASDAI-50 (40.5 vs. 19.1%, *p* < 0.01) and ASDAS responses of inactive disease (17.4 vs. 1.7%, *p* < 0.001), major improvement (27.9 vs. 8.7, *p* < 0.01), and clinically important improvement (53.5 vs. 28.7%, *p* < 0.01) at week 24. Improvements in axial symptoms were observed irrespective of HLA-B27 status (Table 1) (Helliwell et al., 2020). No RCTs were conducted on axial-SpA.

*Risankizumab* (BI 655066/ABBV-066) is a humanized IgG1 MoAb that selectively inhibits IL-23 by specifically targeting the p19 subunit 18. It is approved for the treatment of moderate to severe psoriasis (Mease et al., 2018b). In a phase II RCT, it showed efficacy in the treatment of PsA, but no data are specifically reported on axPsA patients. In another phase II RCT, risankizumab failed to meet the study's primary endpoint and showed no evidence of clinically meaningful improvements compared with placebo in patients with active AS (Baeten et al., 2018).

### Janus Kinase Inhibitors

*Tofacitinib* is a JAK1/JAK3 inhibitor approved for the treatment of PsA. Two phase III RCTs (ORAL-Beyond and ORAL Broaden) demonstrated the efficacy of tofacitinib in the treatment of peripheral involvement, but the effect on the axial domain was not specifically investigated (Gladman et al., 2017). In a phase II RCT in AS bio-naïve patients (<4% with PsO), tofacitinib 5 and 10 mg twice daily demonstrated greater clinical efficacy vs. placebo in reducing signs, symptoms, and objective endpoints of active AS in adult patients with a similar 12-week safety profile as reported in other indication (van der Heijde et al., 2017). A *post-hoc* analysis on the same study showed that approximately one-third of tofacitinib-treated AS patients experienced clinically meaningful reductions in spinal MRI inflammation at week 12. Patients achieving myelin imaging compound for MRI inflammation had a greater clinical response (Maksymowych et al., 2018).

*Upadacitinib* is another selective JAK1 inhibitor. It is approved for the treatment of PsA and recently also for AS. In two phase III RCTs (SELCET PsA1 and PsA2) in PsA with peripheral arthritis, upadacitinib was demonstrated effective in the achievement of ACR20, 50, and 70 (Genovese et al., 2020; Mcinnes et al., 2020). In a *post-hoc* analysis of these two studies, involving ~400 PsA patients with physician-diagnosed spondylitis, treatment with UPA 15 and 30 mg resulted in significantly greater improvements from baseline in the overall BASDAI, BASDAI questions #2 (neck/back/hip pain) and #3 (joint swelling/pain), and ASDAS-CRP endpoints at weeks 12 and 24 vs. placebo. Similarly, significantly higher percentages of pts on UPA 15 and 30 mg achieved BASDAI 50, ASDAS ID, LDA, MI, and CII at weeks 12 and 24 vs. placebo (Table 1) (Deodhar et al., 2020c). The effectiveness of upadacitinib was reported in phase II/III RCT on AS, where data on psoriasis were not reported (van der Heijde et al., 2019).

*Filgotinib* is a selective JAK1 inhibitor. In a phase II RCT (EQUATOR) on 131 PsA patients with peripheral arthritis, 80% in the filgotinib group vs. 33% in the placebo group achieved ACR20 at week 16 (*p* < 0.0001) (Mease et al., 2018a). No data on axial involvement were reported. In a phase II RCT (TORTUGA) on 116 patients with AS, the mean ASDAS change values from baseline to week 12 were −1.47 in the filgotinib group and −0.57 in the placebo group, with an LS mean difference between groups of −0.85 (95% CI −1.17 to −0.53; *p* < 0.0001) (Mease et al., 2018a).

*Deucravacitinib* is a novel oral agent that selectively inhibits tyrosine kinase 2. In a phase II RCT, it demonstrated efficacy in peripheral PsA, but there are no data on axial disease (Mease et al., 2020).

## Discussion

The results of this review suggest that, although the interest in the axial disease in PsA has progressively increased in the last years, specific and high-quality evidence on the efficacy of the current targeted therapies in this subset of patients still represents an unmet need.

Almost all PsA patients recruited in clinical trials had peripheral arthritis, and only a small number had axial involvement. Only one RCT was specifically designed to assess the efficacy of a b-DMARD in axPsA, demonstrating significant improvements across multiple clinical and imaging endpoints in a population with high activity of inflammatory back pain treated with secukinumab. For other b- and ts-DMARDs (ixekizumab, ustekinumab, guselkumab, and upadacitinib), the most specific evidence for potential efficacy in axPsA treatment are derived from *post-hoc* analysis of RCTs based on PsA patients with concomitant peripheral and axial involvement.

Current studies on axPsA are affected by major limitations, starting from the lack of a validated and widely accepted definition of axPsA that prevents identifying a homogeneous group of patients in which reliable clinical trials can be performed. This is why a steering committee including members from the Group for Research and Assessment of Psoriasis and Psoriatic Arthritis and the Assessment of SpA International Society is working toward developing an evidence-based and widely accepted definition of axPsA (the AXIS trial) (Gladman et al., 2021). A further major limitation in the studies on axPsA is that in the absence of specific outcome measures for axPsA, all studies have borrowed assessment tools from AS (i.e., BASDAI and ASDAS). However, such instruments are not validated for axial disease in PsA, and the burden of peripheral arthritis may impact them. Indeed, improvements in other domains may result in an overall improvement of such outcome measures, even if there are no significant changes in the axial disease activity. This could partially explain the conflicting evidence regarding ustekinumab, which demonstrated efficacy in a *post-hoc* analysis on axPsA but not in an RCT in AS, or it may explain why guselkumab was found to be useful in axPsA, but risankizumab, another IL-23 inhibitor, did not meet the primary endpoint in AS patients. Finally, extremely poor data are available on pharmacogenetic of PsA, particularly for axPsA. Although substantive advances have been made in the understanding of genetics in psoriatic disease over the last decade, we are still at an early stage. The rapid emergence of affordable high-throughput technology will likely lead to the discovery of genetic factors that may lead to a more in-depth comprehension of mechanisms underlying the different phenotypical patterns of PsA and more precise profiling of axPsA patients. Such findings may hopefully support clinicians in choosing for each patient the treatment with higher likelihood efficacy (Rahmati et al., 2020).

In conclusion, there is an urgent need for more reliable data derived from studies specifically designed for axPsA and based on a validated definition of axPsA and on specific outcome measures. Moreover, in the prospective of personalized medicine, a further effort should also be made to develop specific studies aimed to identify genetic biomarkers of axPsA.

## Author Contributions

AF and AC conceptualized the review. AF, MC, EC, and MA contributed to analyzing the data and wrote the manuscript. AC and MP supervised the work. All authors reviewed the article and approved the submitted version.

## Conflict of Interest

The authors declare that the research was conducted in the absence of any commercial or financial relationships that could be construed as a potential conflict of interest. The handling editor declared a past co-authorship with the authors MP and AC.
